# 

**Published:** 1860-09

**Authors:** 


### LAGER BEER,

### CONTENTS OF HALL'S JOURNAL OF HEALTH FOR AUGUST.

(New-York, $1 a year.)

HOUSEHOLD SLAVERIES, .................... 193
MIASM, .................... 204
NIGHT AIR, .................... 213
LAGER BEER, .................... 216

